# Supplementary material for: Rbpms2 promotes female fate upstream of the nutrient sensing Gator2 complex component Mios
Source: Nat Commun. 2024 Jun 19;15:5248. doi: 10.1038/s41467-024-49613-2 (PMC11187175; doi:10.1038/s41467-024-49613-2)
Supplement: Supplementary file 1 — Supplementary Information [file 41467_2024_49613_MOESM1_ESM.pdf]

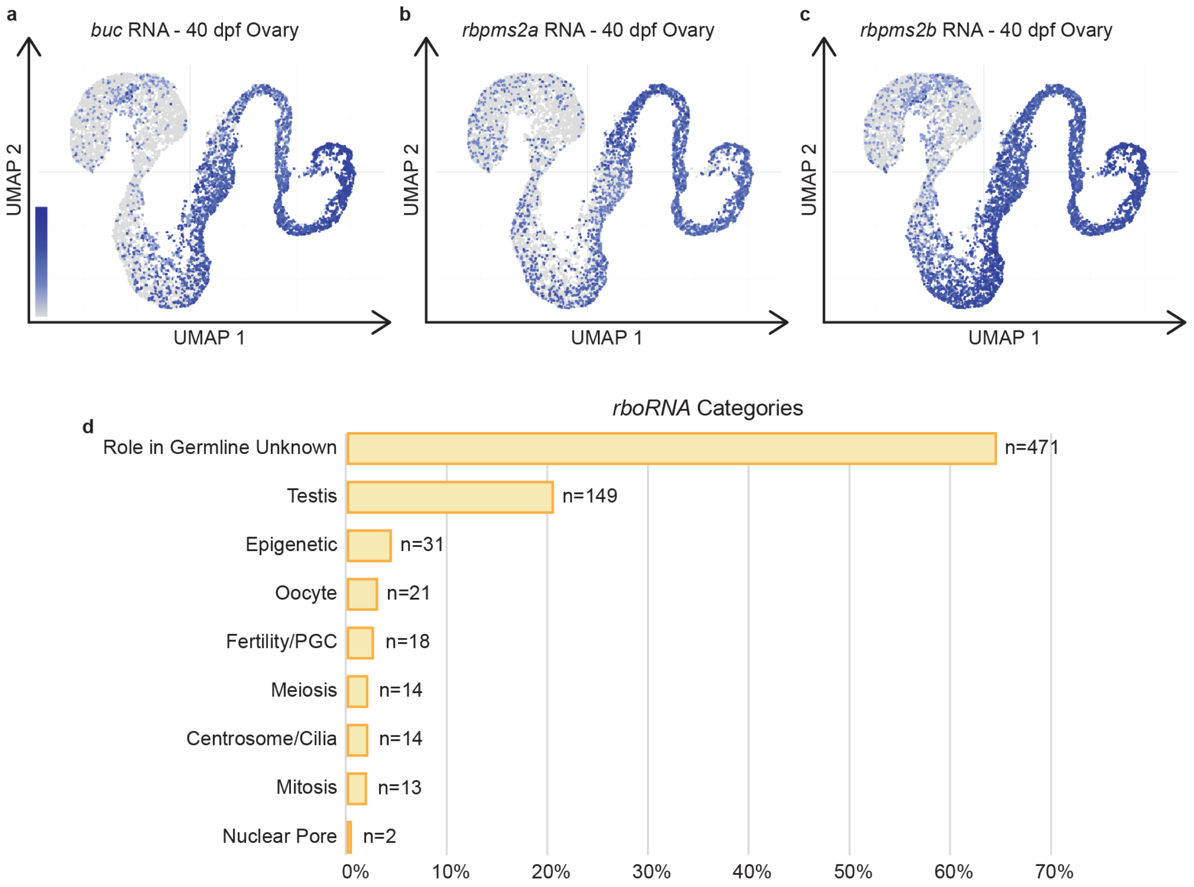

Supplemental Figure 1. Several *rboRNAs* are associated with testis fates. (a-c) UMAPs of *buc*, *rbpms2a*, and *rbpms2b* in the 40 dpf ovary. (d) Categorization of *rboRNAs* by literature search. Number of *rboRNAs* in each category are indicated. Source data are provided as a Source Data file.

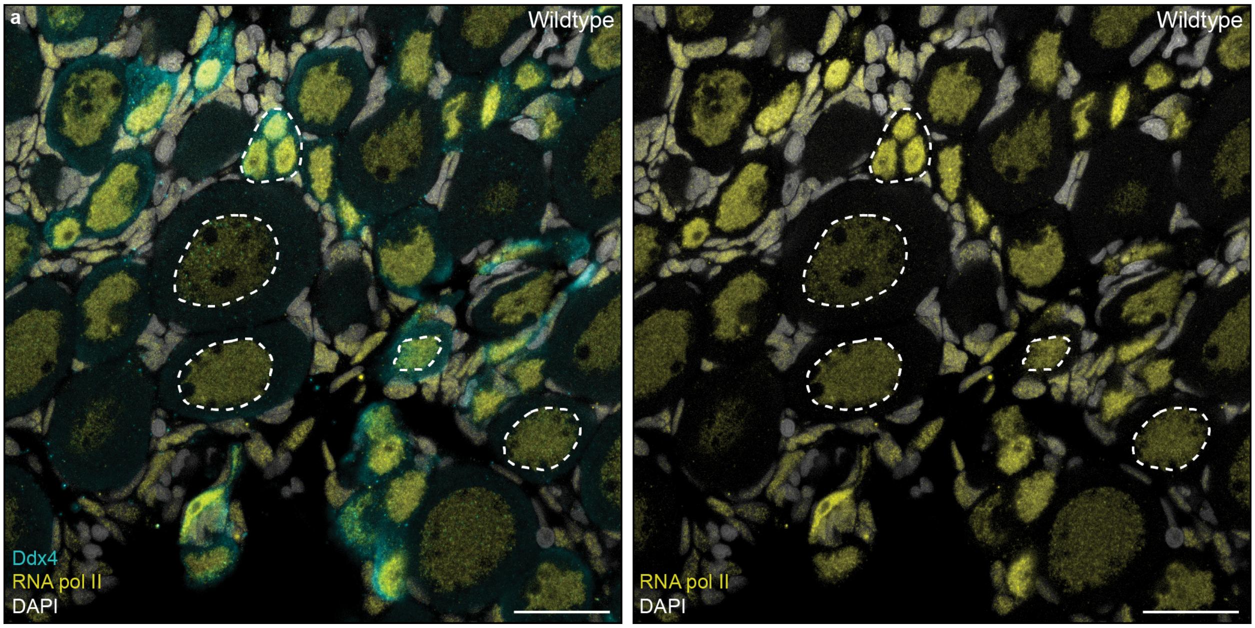

Supplemental Figure 2. RNA pol II localization in gonocytes and prophase I oocytes. (a) RNA pol II (yellow) localization in 35 dpf wildtype (n=4) gonads. Ddx4 labels germ cells (teal) and DAPI labels nuclei (white). Dashed white lines indicate nuclei of different cells stages and adjacent panel shows RNA pol II and DAPI localization in indicated cells. Scale bar is 50 μM.

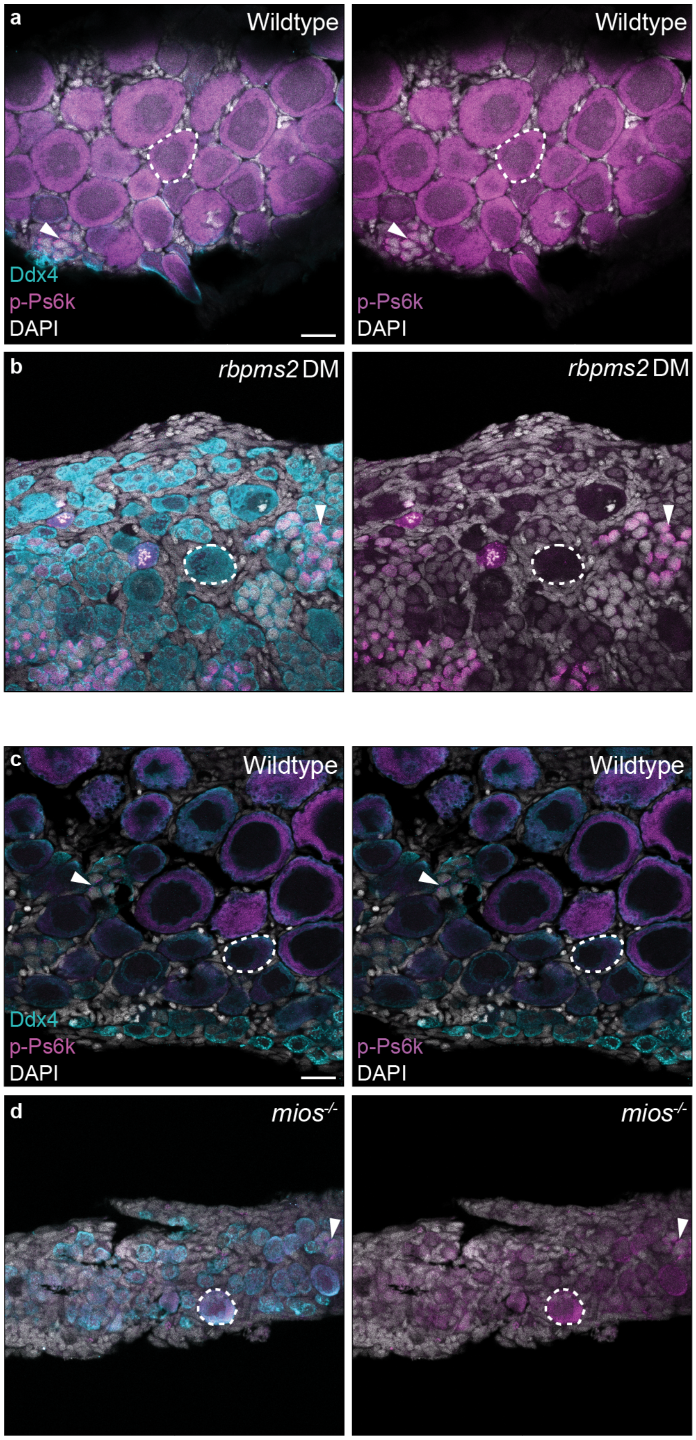

Supplemental Figure 3. mTorc1 signaling in *rbpms2* DMs and *mios*$^{-/-}$. (a-b) Immunostaining of p-Ps6k (purple) in 29-33 dpf wildtype (*rbpms2a*^{ae30}*; rbpms2b*^{sa9329} heterozygous mutant; n=4) and *rbpms2* DM (*rbpms2a*^{ae30}*; rbpms2b*^{sa9329} double homozygous mutant; n=4) germ cells. (c-d) Immunostaining of p-Ps6k (purple) in 35 dpf wildtype (n=7) and *mios*$^{-/-}$ (n=10) germ cells. Germ cells are labelled with Ddx4 (teal)

and nuclei are labelled with DAPI (white). (a, c) Arrows indicate p-Ps6k localization in mitotic and early meiotic nuclei. (b, d) Dashed lines indicate early oocytes. Adjacent panels show p-Ps6k and DAPI localization in indicated cells. Scale bars are 20 μM.

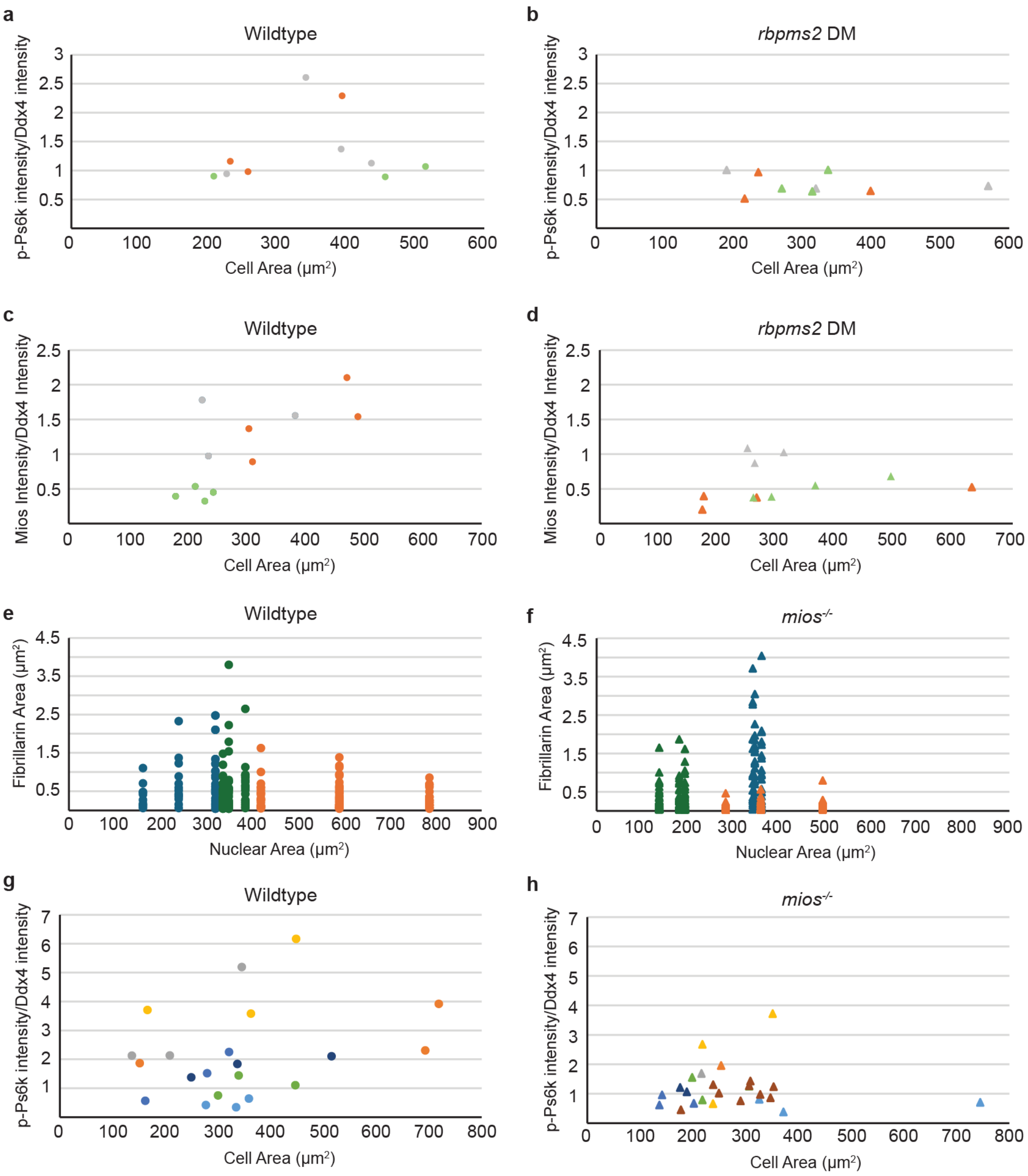

Supplemental Figure 4. Quantification of p-Ps6k, Mios, and Fibrillarin in control and mutant oocytes. (a-b, g-h) Distribution of p-Ps6k fluorescence intensity normalized to Ddx4 fluorescence intensity and correlated to (a-b) wildtype and *rbpms2* DMs and (g-h) wildtype and *mios*$^{-/-}$ oocyte size. (a; g) Circles indicate individual cells (a, n=10 cells; g, n=21 cells) and colors correspond to the specific fish (a, n=3 fish; g, n=7 fish). (b; h) Triangles indicate individual cells (b, n=9 cells; h, n=25 total) and colors correspond to the specific fish (b, n=3 fish; h, n=8 fish). (c-d) Distribution of Mios fluorescence intensity normalized to Ddx4 fluorescence intensity and correlated to wildtype and *rbpms2* DMs oocyte size. (c) Circles indicate individual cells (n=11 cells) and colors correspond to the

specific fish (n=3 fish). (d) Circles indicate individual cells (n=11 cells) and colors correspond to the specific fish (n=3 fish). (e-f) Distribution of Fibrillarin puncta area and nuclear area of a given oocyte in wildtype and *mios*$^{-/-}$ fish. (e) Circles indicate individual cells (n=9 cells) and colors correspond to the specific fish (n=3 fish). (f) Circles indicate individual cells (n=9 cells) and colors correspond to the specific fish (n=3 fish). Source data are provided as a Source Data file.

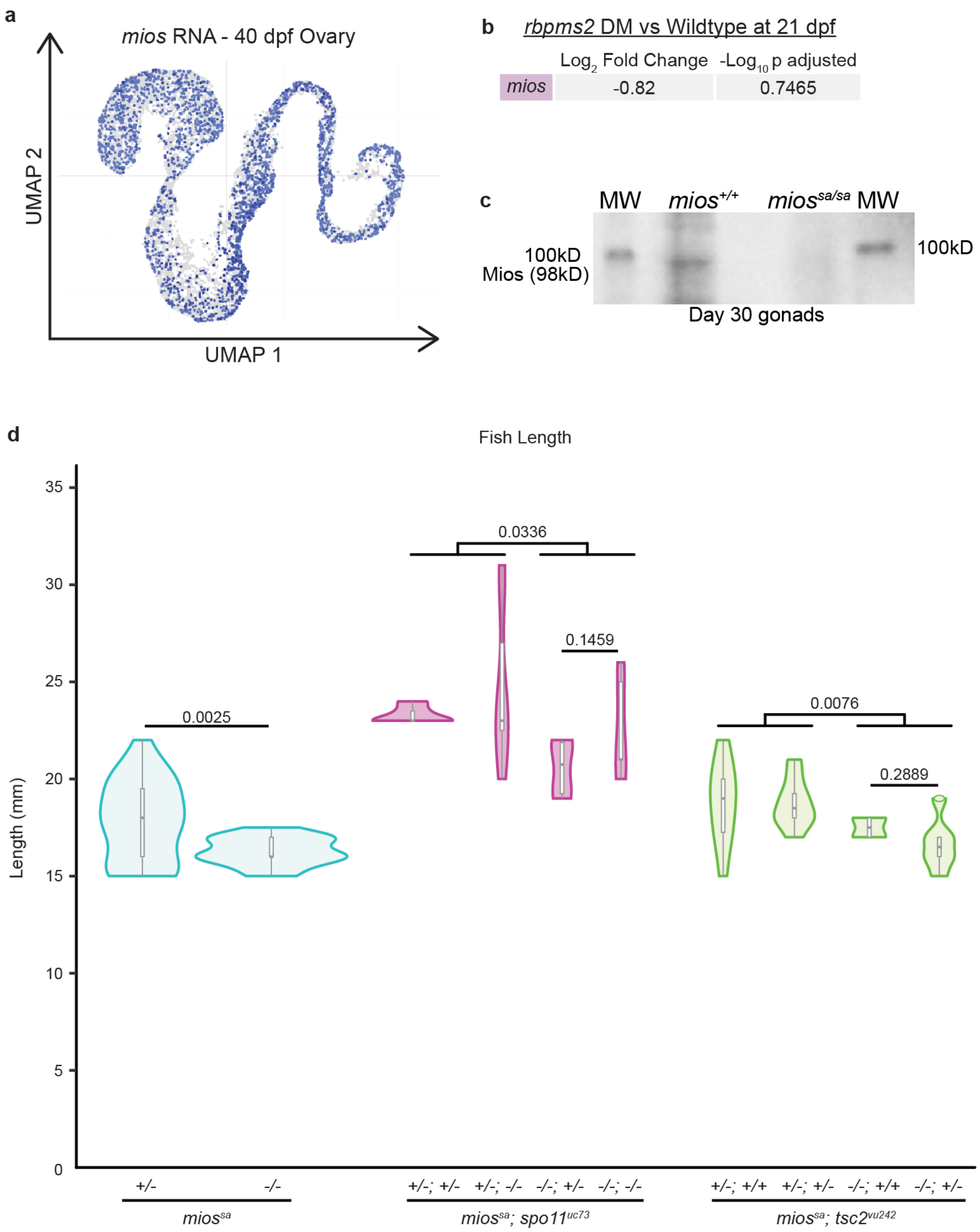

Supplemental Figure 5. Mios is absent in *$mios^{sa/sa}$* fish and loss of Mios delays fish growth. (a) UMAP of *mios* in the 40 dpf ovary[1]. (b) Log$_2$ Fold Change and -Log$_{10}$ p adjusted values of *mios* RNA expression from bulk RNA sequencing of *rbpms2* DMs versus wildtype gonads at 21 dpf. (c) Western blot of Mios in 30 dpf wildtype and *$mios^{sa/sa}$* gonads (repeated twice). (d) Lengths of *$mios^{sa}$* (+/- n=35, -/- n=20), *$mios^{sa}$; $spo11^{uc73}$* (+/-; +/- n=5, +/-; -/- n=7, -/-; +/- n=6, -/-; -/- n=4), and *$mios^{sa}$; $tsc2^{vu242}$* (+/-; +/+ n=10, +/-; +/- n=8, -/-; +/+ n=4, -/-; +/- n=6) fish. For box and whisker plots, line indicates median, boxes indicate upper (75th) and lower quartiles (25th), whiskers indicate data

within 1.5 times the inner quartile range, and error bars represent minimum and maximum values. Any data outside of this range are plotted as individual points. Two-tailed paired equal variance student's t tests were performed for the indicated groups. *P*=0.05. Source data are provided as a Source Data file.

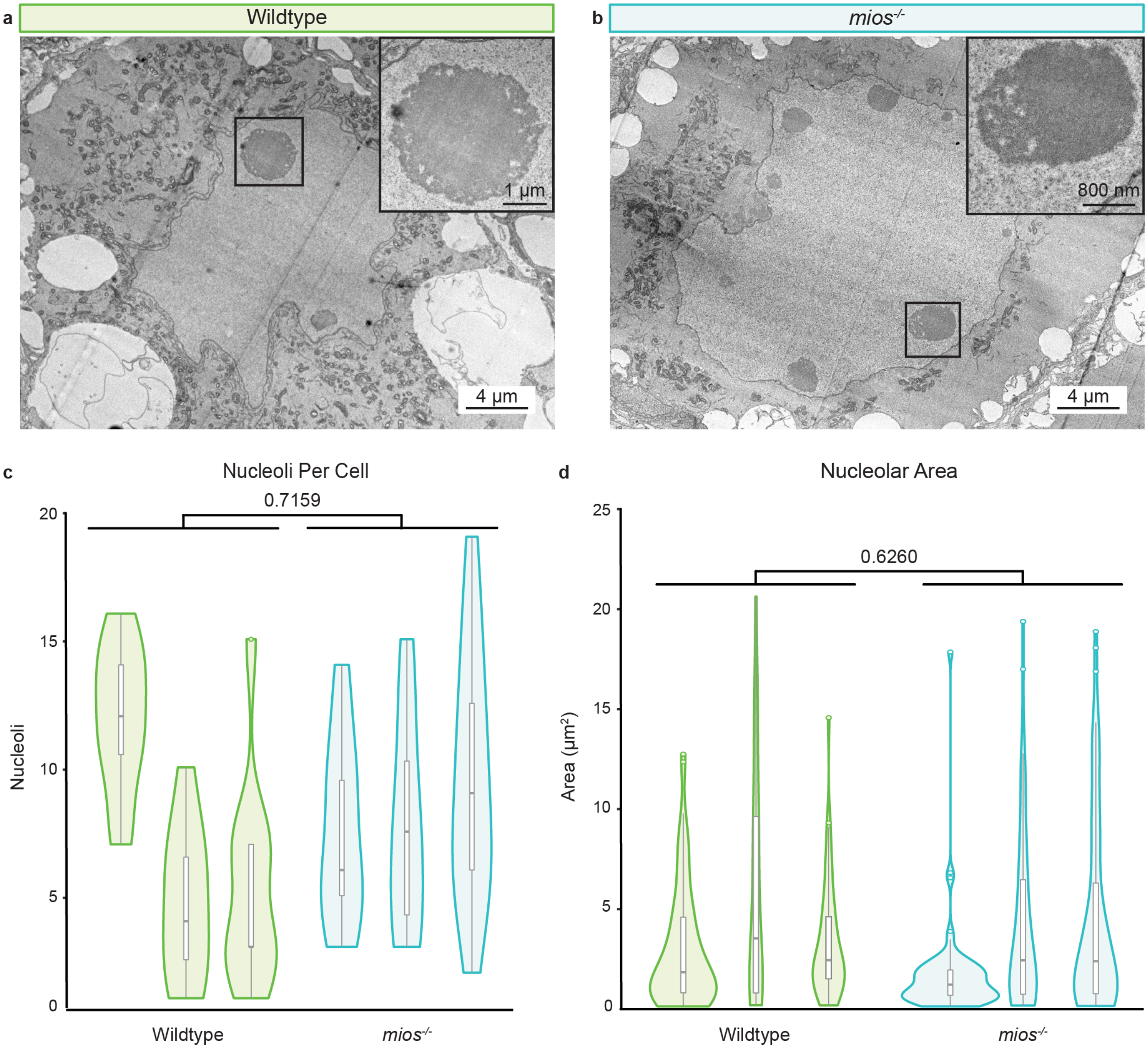

Supplemental Figure 6. Nucleoli do not show significant ultrastructural differences between wildtype and *mios*$^{-/-}$ oocytes. (a-b) Transmission electron micrographs of 35 dpf wildtype and *mios*$^{-/-}$ oocytes. Inset shows representative nucleolus for each genotype and corresponding scale bar. (c) Quantification of nucleoli per cell for 35 dpf wildtype (n=3) and *mios*$^{-/-}$ (n=3) oocytes. (d) Quantification of nucleolar area from 35 dpf wildtype (n=3) and *mios*$^{-/-}$ (n=3) oocytes. (c-d) Nucleoli were analyzed from wildtype (n=11 cells per fish) and *mios*$^{-/-}$ (n=11, 10, and 14 for each fish, respectively) oocytes. For box and whisker plots, line indicates median, boxes indicate upper (75th) and lower quartiles (25th), whiskers indicate data within 1.5 times the inner

quartile range, and error bars represent minimum and maximum values. Any data outside of this range are plotted as individual points. Two-tailed paired equal variance student’s t tests were performed for the indicated groups. *P*=0.05. Source data are provided as a Source Data file.

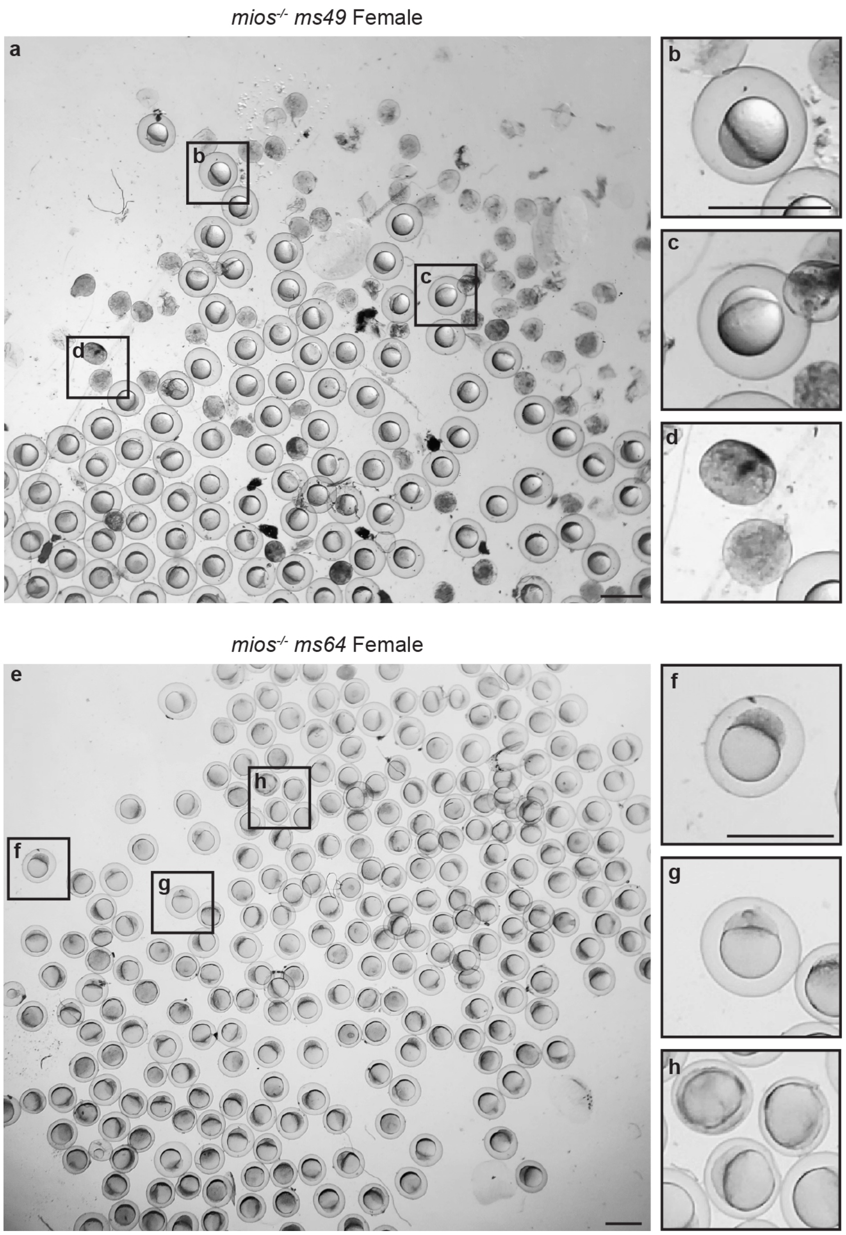

Supplemental Figure 7. *mTOR^{ca} ms49* and *ms64 mios$^{-/-}$* egg phenotypes. (a) Representative eggs from a *mios$^{-/-}$ mTOR^{ca} ms49* female and (e) representative eggs from a *mios$^{-/-}$ mTOR^{ca} ms64* female. (b, f) Representative activated, fertilized egg. (c, g) Representative unfertilized egg. (d) Representative degenerating eggs. (h) Representative eggs with activation deficits. Scale bar is 1mm.

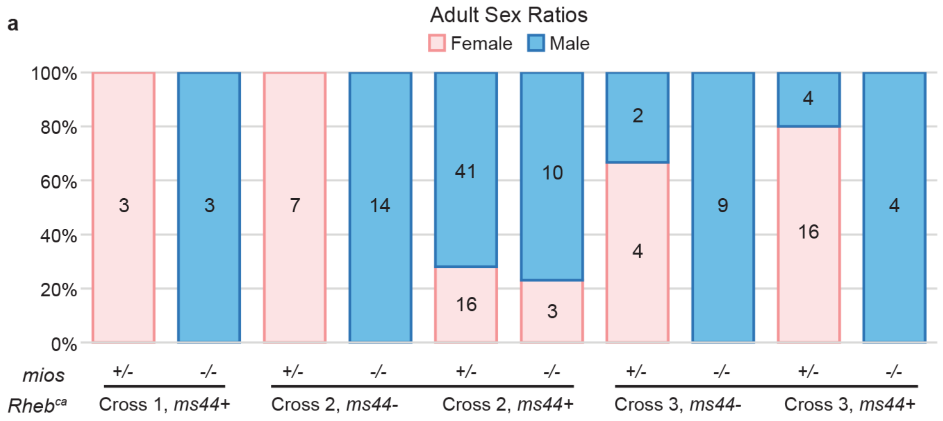

Supplemental Figure 8. Incompletely penetrant suppression of *mios-/-* phenotypes by the *Rheb*^{ca} *ms44* allele. (a) Sex ratio graph for 60 dpf+ fish with the *ms44 Rheb*^{ca} allele from three independent crosses. Pink represents female and blue represents males; number of fish screened are indicated on the bars for each group. Source data are provided as a Source Data file.

Supplemental Table 1. Guide RNAs and Genotyping Primers.

Oligo Name	Sequence (5' to 3')
mios sgRNA_1	atttaggtgacactataGAGCTCAGTCTGTACCGGATgttttagagctagaaatagcaag
mios sgRNA_2	atttaggtgacactataAGGCCACGCATTTCATAAAGgttttagagctagaaatagcaag
mios sgRNA_1 and 2_F	CCAGATATCCTGTGGTCTCCTC
mios sgRNA_1_R1	CCACAGCTAACAAACACTCTGG
mios sgRNA_1_R2	CACAGCTAACAAACACTCTGGC
mios sgRNA_2_R1	CCTTACTTTTGGAGTTGTGGCT
mios sgRNA_2_R2	TGTCCCACAGCTAACAAACACT
mios cDNA_F	ACAAGCCACTGTCCTGCC
mios cDNA_R	TACGCTATCTGAAGGCACCAG
mios ms20 F	CCAGATATCCTGTGGTCTCCTC
mios ms20 R	CATTGGTATGTCCCACAGCTAA
mios sa F	GCCGAGTCGTGTTAACCACTT
mios sa R1	GCCAGCCAGTTACTGTCCAC
mios sa R2	ACGTCTTCTGGCTGGTGTTA
rbpms2a ae30 F	TTTGCTAAAGCCAACACGAA
rbpms2a ae30 R	ATTCACCCTGGCCAGAGTTT
rbpms2b sa F_Mutant Assay	CACTTATCAAGCTAACTTCAAAGCAGA
rbpms2b sa F_Wildtype Assay	TTTCAGGGTTATGAGGGTTCA
rbpms2b sa R	GCCAAAAGCAAAATATCAAACA
spo11 uc73 F	TCACAGCCAGGATGTTTTGA
spo11 uc73 R	CACCTGACATTGTTCCAGCA
tsc2 vu242 F	CCAGCACCACCTGCAGTCTGG
tsc2 vu242 R	CTCTTGGGCAGAGCAGAGAAGTTGG
